# Editorial: The placenta: the origin of chronic diseases in adults

**DOI:** 10.3389/fendo.2024.1455965

**Published:** 2024-07-11

**Authors:** Fernanda R. Giachini, Deanne H. Hryciw, Mauricio Castro-Parodi, Alicia E. Damiano

**Affiliations:** ^1^ Institute of Biological and Health Sciences, Federal University of Mato Grosso, Barra do Garças, Brazil; ^2^ Griffith Institute of Drug Discovery, Griffith University, Brisbane, QLD, Australia; ^3^ Universidad de Buenos Aires, Facultad de Farmacia y Bioquímica, Depto. de Ciencias Biológicas, Cátedra de Biología Celular y Molecular, Buenos Aires, Argentina; ^4^ Universidad de Buenos Aires-Consejo Nacional de Investigaciones Científicas y Técnicas (CONICET), Instituto de Fisiología y Biofísica Bernardo Houssay (IFIBIO Houssay), Buenos Aires, Argentina

**Keywords:** placenta, chronic diseases, metabolic diseases, offspring, epigenomic, fetal programming

Traditionally viewed as a temporary interface between mother and fetus, the placenta is currently recognized as a dynamic organ with far-reaching effects on the long-term health of the growing fetus. This organ mediates the exchange of nutrients, oxygen, and waste products, playing a pivotal role in fetal growth and development (1). Consequently, the placenta is susceptible to various insults such as maternal stress, infections, and metabolic disturbances, which can alter its structure and function.

An emerging area of research worldwide is the fact that the impact of perturbations in placental function is not limited to intrauterine life or newborns, but can persist for years, potentially contributing to disease conditions in adulthood. Numerous studies have established links between adverse placental conditions, such as impaired nutrient transport or inflammation, and an elevated risk of cardiovascular disorders, diabetes, and neurological conditions (2–4). These associations underscore how the quality of the intrauterine environment, heavily influenced by placental health, can predispose individuals to health challenges later in life, and much effort must be directed to elucidate this still unclear relationship.

Over the past five decades, the concept of “developmental origins of health and disease” (DOHaD) has evolved, linking early environmental factors with health and disease risks from childhood through adulthood (5). This interdisciplinary field encompasses epidemiology, clinical research across various specialties, public and global health, experimental physiology, molecular biology (especially epigenetics), developmental biology, anthropology, social sciences, and evolutionary biology. Fetal programming, central to the DOHaD concept, posits that the fetus adjusts its developmental trajectory in response to signals from the maternal environment during pregnancy. This adaptive process is believed to optimize the fetus’s chances of survival and health by responding to environmental cues.

In light of these concepts, the Research Topic entitled “The Placenta: The Origin of Chronic Diseases in Adults” aimed to compile original research articles and reviews highlighting recent advances in understanding placental functions and their profound impacts on the health outcomes of mothers and offspring. In this manner, various research groups presented their perspectives and innovative data on topics related to the theme proposed for this Research Topic.

Understanding the intricate biological processes controlled by the placenta is challenging due to its dynamic nature and the variety of functions it performs. Studies on fetal programming emphasize how prenatal environmental influences, such as intrauterine food restriction (IFR), can lead to low birth weight and predispose offspring to adult obesity and metabolic disorders. In this Research Topic, Andreotti et al. highlighted the effects of IFR on adipose tissue metabolism in offspring, underscoring the long-term health implications of early developmental influences.

Maternal stress during pregnancy is widely accepted to correlate with adverse outcomes for the fetus (6). Consequently, variations in the placental microbiome, exposure to toxins, maternal metabolic disorders, and placental abnormalities can induce epigenetic changes that impact health across the lifespan. In this regard, Ruiz-Triviño et al. reviewed the impact of maternal microbiota on fetal programming, emphasizing its role in influencing fetal development and postnatal health outcomes. Dysbiosis in maternal gut microbiota may contribute to adult morbidity, illustrating the interconnectedness of maternal health and lifelong well-being.

Pregnancies complicated with Gestational Diabetes Mellitus and preeclampsia underscore how these conditions contribute to maternal stress, affecting epigenetic processes and posing risks for both mothers and newborns. In this field, Gomez Ribot et al. explored the benefits of an extra virgin olive oil-enriched diet in managing maternal insulin resistance and enhancing placental metabolism, potentially benefiting fetal development. Research efforts also are focused on identifying biomarkers for predicting gestational dysfunctions.

Current technologies may not be advanced enough to fully capture the nuances of placental function and its impact on long-term health. Advancements in imaging, molecular biology, and data analysis are needed. Thinking on this matter, Ma et al. utilized bioinformatics to discover potential markers linked to placental immune tolerance, enhancing understanding of the underlying molecular mechanisms of preeclampsia and identifying four candidate genes (ANKRD37, CRH, LEP, SIGLEC6) as potential diagnostic targets.

Isolating the specific impact of placental function on later health outcomes is complicated by the multifactorial nature of diseases. Numerous factors, including genetics, postnatal environment, and lifestyle choices, interact with placental health, opening the possibility to the investigation of epigenetics in the placental tissue. Concerning this topic, Gu et al. investigated histone H3K9 hypermethylation in placental trophoblasts from preeclamptic pregnancies, revealing insights into oxidative stress-induced epigenetic modifications that affect placental function and fetal programming.

In conclusion, the placenta plays a key role in fetal development, with an impact that extends beyond pregnancy. Its significant influence on adult health highlights the critical importance of prenatal care and maternal health in shaping the well-being of future generations. Investigating the long-term effects of placental function on adult health requires extensive longitudinal studies, which are time-consuming, costly, and require sustained funding and participant retention over many years. However, by unraveling the complexities of placental health and its broad implications, we can envision innovative strategies for preventing diseases and promoting health throughout every stage of life.

## Author contributions

FG: Writing – review & editing. DH: Writing – review & editing. MC-P: Writing – review & editing. AD: Writing – original draft, Writing – review & editing.
